# Determinants of COVID-19 vaccination worldwide: WORLDCOV, a retrospective observational study

**DOI:** 10.3389/fpubh.2023.1128612

**Published:** 2023-08-31

**Authors:** Alberto Peano, Gianfranco Politano, Maria Michela Gianino

**Affiliations:** ^1^Department of Public Health Sciences and Pediatrics, University of Turin, Turin, Italy; ^2^Department of Control and Computer Engineering, Polytechnic of Turin, Turin, Italy

**Keywords:** COVID-19 vaccine, global health, health policy, vaccination coverage, vaccination policy, determinants of vaccination, international health, public health

## Abstract

**Introduction:**

The COVID-19 pandemic has resulted in numerous deaths, great suffering, and significant changes in people's lives worldwide. The introduction of the vaccines was a light in the darkness, but after 18 months, a great disparity in vaccination coverage between countries has been observed. As disparities in vaccination coverage have become a global public health issue, this study aimed to analyze several variables to identify possible determinants of COVID-19 vaccination.

**Methods:**

An ecological study was conducted using pooled secondary data sourced from institutional sites. A total of 205 countries and territories worldwide were included. A total of 16 variables from different fields were considered to establish possible determinants of COVID-19 vaccination: sociodemographic, cultural, infrastructural, economic and political variables, and health system performance indicators. The percentage of the population vaccinated with at least one dose and the total doses administered per 100 residents on 15 June 2022 were identified as indicators of vaccine coverage and outcomes. Raw and adjusted values for delivered vaccine doses in the multivariate GLM were determined using R. The tested hypothesis (i.e., variables as determinants of COVID-19 vaccination) was formulated before data collection. The study protocol was registered with the grant number NCT05471635.

**Results:**

GDP per capita [odds = 1.401 (1.299–1.511) CI 95%], access to electricity [odds = 1.625 (1.559–1.694) CI 95%], political stability, absence of violence/terrorism [odds = 1.334 (1.284–1.387) CI 95%], and civil liberties [odds = 0.888 (0.863–0.914) CI 95%] were strong determinants of COVID-19 vaccination. Several other variables displayed a statistically significant association with outcomes, although the associations were stronger for total doses administered per 100 residents. There was a substantial overlap between raw outcomes and their adjusted counterparts.

**Discussion:**

This pioneering study is the first to analyze the association between several different categories of indicators and COVID-19 vaccination coverage in a wide complex setting, identifying strong determinants of vaccination coverage. Political decision-makers should consider these findings when organizing mass vaccination campaigns in a pandemic context to reduce inequalities between nations and to achieve a common good from a public health perspective.

## 1. Introduction

The COVID-19 pandemic has had major health, economic, and social implications (1, 2). The COVID-19 pandemic resulted in a considerable number of deaths due to the highly contagious nature of the virus (3, 4). As a result, the COVID-19 pandemic has become a sudden, widespread, and urgent public health issue.

Initially, when faced with a new and unknown aetiopathological entity (5), different countries adopted different policies for containing COVID-19 contagion that turned out to be more or less effective (6, 7). While social and hygiene measures (i.e., so-called social distancing, curfews, the use of masks and hand hygiene) initially had a positive impact on reducing infections (8), for the most part, they proved to be ephemeral measures. These actions were short-lived because they were not sustainable over time due to the damage they caused to mental health (9) and the low adherence of the population (10, 11). The relaxation of these restrictive measures consistently led to an increase in the spread of the COVID-19 virus, with a resurgence of hospital admissions, an ever-increasing occupancy of intensive care beds, and a subsequent increase in the number of deaths.

Since the beginning of the COVID-19 pandemic, researchers have been working to enable the identification of the SARS-CoV-2 viral genome and possible target proteins for treatment. The rapid development of several candidate vaccines using different vaccine technologies and subsequent clinical testing was possible (12) owing to years of vaccine research and the launch of the Access to COVID-19 Tools (ACT)–Accelerator partnership (13).

Additionally, due to the regulatory agencies' solicitude, in certain countries (e.g., Israel, the UK, the US, and the EU), the COVID-19 vaccination campaign started at the end of 2020 and extended worldwide during 2021 (14) [apart from Eritrea and the Democratic Republic of Korea (15, 16)].

Through purchasing agreements with individual vaccine manufacturers, governments (or supranational institutions, e.g., the EU) secured the supply of the necessary doses. However, this entailed a division of the world's population depending on the negotiating power of the country of residence and, therefore, its economic strength. COVAX is one of the three pillars of the ACT-Accelerator programme that sought to remedy this situation: Dedicated to vaccines, the purpose of COVAX is to accelerate the development and manufacture of COVID-19 vaccines, ensuring fair and equitable access for every country in the world (17, 18). However, COVAX cannot distribute all doses received without delays (19).

Approximately 24% of the world's population had received at least one dose of vaccine by mid-2021. However, only 1% of people in low- and lower-middle-income countries were vaccinated, reflecting inequalities within the global health order, an extension of historical political and economic differences (19).

Data from the University of Oxford's Our World in Data database (20) show that 67.7% of the world population has received at least one dose of a COVID-19 vaccine, 12.57 billion doses have been administered globally, and 4.88 million are being administered daily. The data reveal large differences in vaccination rates between countries: as of 30 August 2022, the share of people vaccinated against COVID-19 ranged from over 95 per 100 in Qatar to less than 20% in Nigeria and Congo, and the cumulative number of doses administered per 100 people ranged from 365 per 100 in Cuba to under 50 doses per 100 in countries in several African states. These data highlight the importance of understanding the numerous factors affecting these differences in vaccination coverage.

This study aimed to identify potential determinants among numerous variables in multiple fields—sociodemographic, cultural, infrastructural, economic, political, and health system performance—associated with vaccination coverage through an analysis of two indicators of vaccination coverage (outcomes) across 205 nations worldwide.

## 2. Methods

### 2.1. Study design and setting

The Worldwide Determinants of COVID-19 Vaccination (WORLDCOV) study was a retrospective observational study conducted using pooled secondary data.

### 2.2. Participants

All countries (independent or not) worldwide for which vaccination data were available and variables of interest were accessible were included. If no data on vaccination coverage and the dose-administered population ratio were available or a substantial lack of data had been reported, the country was excluded from the analysis.

At the end of the recruitment phase, 205 countries and territories were included and are listed in Supplementary Table S1.

### 2.3. Outcomes, variables, and data sources

Two outcomes were identified as indicators of vaccination coverage: the percentage of the population vaccinated with at least one dose (that is, the number of people who have received at least one vaccine dose per 100 people in the total population) and the total doses administered per 100 residents (“total doses administered” per 100 people of the total population) (21). The two indicators are complementary because the first indicates the percentage of the vaccinated population (independent of the n. of the doses) and the second allows us to analyze how many vaccine doses have been administered to the population on average. These two analyzes highlight important inequalities in global vaccine access. These data were obtained as of 15 June 2022 for each country. If data were not available on that date, the most recent available data were used for retrospective assessment.

All COVID-19 vaccines approved by national or international regulatory agencies were included, despite the vaccine technology, manufacturer, and regulatory agency that granted marketing authorization.

Several variables were accounted for to analyze whether they were, and which of them were, determinants of vaccination coverage, considering the 2020 value for each country or the most recent available figure if the 2020 value was unavailable. The variables considered in the present study are shown in Table 1, which illustrates the field, definition, source, and unit of measure for each of them. Variables were chosen based on prior knowledge (38) [e.g., Greenberg Index as a proxy of ethnocultural differences (28)], precedent literature on COVID-19 (39–43), epidemiological research, and the availability of data.

**Table 1:** Variables, fields, definitions, units of measure, and database sources.

**Field**	**Variables**	**Definition**	**Measure**	**Source**
Outcomes	Total doses administered per 100 residents	Number of doses administered per 100 residents	Percentage of whole population	Our World in Data (20)
	Percentage of the population vaccinated with at least one dose	Population vaccinated with at least one dose	Percentage of whole population	Our World in Data (20)
Sociodemographic	Total population	Country's inhabitants	Number of country's inhabitants	Our World in Data (20)
	Population density	The number of individuals per unit geographic area	Number of people per square kilometer	Our World in Data (20)
	Median age	The median age of the country's inhabitants	Years	Our World in Data (20)
Economic	Gini Index	Measure the extent to which income distribution within a country deviates from a perfectly equal distribution	100 represented full inequality, while 0 the perfect equality	The World Bank, Data (22)
	GDP per capita	Gross domestic product divided by midyear population	Current United States Dollars per inhabitant	The World Bank, Data (23)
Healthcare system performance	COVID-19-specific mortality	Total deaths attributed to COVID-19 per 1,000,000 people. Counts can include probable deaths, where reported	Total deaths per 1,000,000 people	Our World in Data (20)
	Type of health system (public or private)	The predominant type of health system, public or private. Derived from Domestic general government health expenditure (percentage of current health expenditure)	1 if public, 0 if private	The World Bank, Data (24)
	Health personnel—physicians	Number of physicians in relation to inhabitants	Number of physicians/1,000 residents	The World Bank, Data (25)
	Health personnel—nurses and midwives	Number of nurses and midwives in relation to inhabitants	Number of nurses and midwives/1,000 residents	The World Bank, Data (26)
Cultural	Literacy rate	People aged 15 and above who can both read and write with understanding a short, simple statement about their everyday life	Percentage of population aged 15 and above	The World Bank, Data (27)
	Greenberg index	Index of linguistic diversity as a proxy of ethnic and cultural diversity among different population groups. To identify the possible association with the cultural fragmentation of a country led to the choice of δ = 1.00, which identifies even the smallest differences between the population	1 represented the maximum linguistic, ethnic and cultural—diversity, 0 represented no diversity	Erkan Gören (28)
	Presence of a predominant religion	More represented religious faith among inhabitants	Percentage of believers of most represented religion respect to total population	World Religion Database (29)
Infrastructural	Density of road network	Extension of the road network with respect to the country's geographic extension	Kilometers per square kilometer	The World Factbook (30)
				The World Bank, Data (31)
	Access to electricity	Population with access to electricity	Percentage of the whole population	The World Bank, Data (32)
Political	Civil liberties	The civil liberties subattribute denotes the extent to which civil rights and liberties are respected. The five civil liberties subcomponents are freedom of expression, freedom of association and assembly, freedom of religion, freedom of movement, and personal integrity and security, each of which reflects core concepts in the human rights literature	0 (lowest score) to 1 (highest score)	The World Bank, GovData360 (33, 34)
	Political stability and absence of violence/terrorism	Perceptions of the likelihood of political instability and/or politically motivated violence, including terrorism	Units of standard normal distribution, i.e., ranging from ~-2.5 to 2.5	The World Bank, DataBank (35, 36)
	Vaccine doses delivered	Total vaccine doses delivered to each country up to 21 June 2022	Total number of doses delivered	UNICEF, COVID-19 Market Dashboard (37)

This study was exempted by the ethics committee due to the use of online data.

This study followed the Strengthening the Reporting of Observational Studies in Epidemiology (STROBE) guidelines.

The study protocol was registered under the grant number NCT05471635.

### 2.4. Data analysis

A set of multivariate regression analyzes were performed to uncover the roles of investigated determinants and test for possible confounding effects to estimate the percentage of the population vaccinated with at least one dose and the total doses administered per 100 residents. All analyzes were performed using R (44). A multivariate GLM was performed to evaluate the role and weight of each determinant. The likelihood of the percentage of the population vaccinated with at least one dose and the total doses administered per 100 residents due to determinants was reported as odds and corresponding 95% confidence intervals (CIs), with a significance level at a *p*-value of < 0.05. To highlight any possible confounding effect introduced by “delivered vaccine doses,” a second set of GLM analyzes was performed with this variable as a confounder. Thus, we compared odds among the two fitted models to determine the presence of differences.

## 3. Results

The data analyzed demonstrated wide ranges due to the inclusion of almost all countries and territories worldwide. The descriptive statistics for the outcomes and covariates are reported in Table 2.

**Table 2:** Descriptive statistics of outcomes and covariates.

	**Mean**	**Minimum**	**Maximum**
Total vaccine doses delivered	72,464,654	6,200	3,73E+09
Total doses administered per 100 residents	132	0.13	356
Percentage of the population vaccinated with at least one dose	57.6	0.12	125
Median age	30.4	15.1	48.2
Total population	38,248,855	18,174	1,44E+09
Population density	450	0.137	20,547
COVID-19-specific mortality	1,198	3.1	6,395
GDP per capita	17,036	239	1,75,814
Gini Index	37.6	23.2	63
Greenberg Index	0.371	0	0.988
Literacy rate	84.1	5.41	100
Presence of a predominant religion	0.808	0.33	0.999
Density of road network	0.961	0	13
Access to electricity	86.7	7.24	100
Civil liberties	0.572	0.13	0.853
Political stability and absence of violence/terrorism	−0.055	−2.73	1.91
Type of health system (public or private)	0.551	0	1
Health personnel—physicians	1.96	0.023	8.42
Health personnel—nurses and midwives	4.41	0.112	20.2

The table reports the mean values and ranges from minimum to maximum.

Statistically significant and concordant results for almost all determinants were found for both outcomes, indicating the presence of several different variables influencing COVID-19 vaccination (Table 3).

**Table 3:** Synthesis of associations between variables and outcomes.

		**Total doses administered per 100 residents**	**Percentage of the population vaccinated with at least one dose**
Positive association	Strong	Access to electricity	Access to electricity
		GDP per capita	GDP per capita
		Political stability and absence of violence/terrorism	Political stability and absence of violence/terrorism
	Intermediate	Total population	Total population
		Gini index	Gini index
	Weak	Population density	Health personnel—physicians
		Health personnel—physicians	
		Health personnel—nurses and midwives	
Negative associations	Strong	Civil liberties	Civil liberties
	Weak	Greenberg index	Greenberg index
		Presence of a predominant religion	Presence of a predominant religion
		Type of health system (public or private)	Type of health system (public or private)
			Literacy rate
No association		Median age	Median age
		COVID-19-specific mortality	COVID-19-specific mortality
		Literacy rate	Density of road network
		Density of road network	Population density
			Health personnel—nurses and midwives

While comparing the results for both outcomes with their adjusted counterparts, there was a substantial overlap. Indeed, the delivered vaccine doses did not substantially affect the total doses administered per 100 residents [odds = 1.025 (1.005–1.044) CI 95%] and likely did not impact the percentage of the population vaccinated with at least one dose [odds = 1.023 (0.994–1.053) CI 95%].

### 3.1. Total doses administered per 100 residents

The total number of doses administered per 100 residents demonstrated the strongest association. First, the results showed that four determinants are extremely impactful in determining total doses administered: GDP per capita [odds = 1.401 (1.299–1.511) CI 95%], access to electricity and political stability [odds = 1.625 (1.559–1.694] CI 95%], and absence of violence/terrorism [odds = 1.334 (1.284–1.387) CI 95%] are positively associated, while the determinant civil liberties [odds = 0.888 (0.863–0.914) CI 95%] is negatively associated with total doses administered per 100 residents. Thus, the higher the GDP per capita and access to electricity and political stability are, the higher the likelihood that a high number of total doses will be administered per 100 residents, while the more civil liberties increase, the lower the likelihood of this outcome. A statistically significant positive association was also found for the total population [odds = 1.190 (1.157–1.225) CI 95%] and the Gini Index [odds = 1.152 (1.123–1.181) CI 95%], although with lower odds than the aforementioned variables. Population density [odds = 1.054 (1.025–1.083) CI 95%], physicians [per 1,000 people; odds = 1.092 (1.06–1.125) CI 95%], and nurses and midwives [per 1,000 people; odds = 1.066 (1.027–1.105) CI 95%] were positively associated with the outcome, although their impact was very modest in comparison with the previously mentioned variables. The Greenberg Index, the presence of a predominant religion, and type of health system were significantly negatively associated with vaccination coverage, although the strength of the association was weak [odds = 0.903 (0.881–0.925) CI 95%, 0.951 (0.929–0.973) CI 95%, and 0.958 (0.935–0.981) CI 95%, respectively]. Median age, COVID-19 mortality, literacy rate, and density of road networks did not show statistically significant associations (Table 4).

**Table 4:** Odds of variables and total doses administered per 100 residents, both raw (first column) and adjusted (second column).

	**Total doses administered per 100 residents**		**Total doses administered per 100 residents—adjusted**	
	**Odds**	**CI 95%**	**Odds**	**CI 95%**
Median age	0.989	0.942–1.039	0.991	0.943–1.041
Total population	1.190	1.157–1.225	1.174	1.139–1.211
Population density	1.054	1.025–1.083	1.060	1.031–1.091
COVID-19-specific mortality	0.983	0.957–1.009	0.985	0.959–1.011
GDP per capita	1.401	1.299–1.511	1.435	1.328–1.551
Gini Index	1.152	1.123–1.181	1.145	1.116–1.175
Greenberg Index	0.903	0.881–0.925	0.902	0.880–0.924
Literacy rate	0.961	0.914–1.011	0.966	0.919–1.017
Presence of a predominant religion	0.951	0.929–0.973	0.956	0.934–0.979
Density of the road network	0.993	0.946–1.042	0.931	0.867–0.999
Access to electricity	1.625	1.559–1.694	1.616	1.550–1.685
Civil liberties	0.888	0.863–0.914	0.898	0.871–0.925
Political stability and absence of violence/terrorism	1.334	1.284–1.387	1.331	1.281–1.384
Type of health system (public or private)	0.958	0.935–0.981	0.954	0.931–0.978
Health personnel—physicians	1.092	1.060–1.125	1.087	1.054–1.120
Health personnel—nurses and midwives	1.066	1.027–1.105	1.063	1.024–1.102

### 3.2. Percentage of the population vaccinated with at least one dose

The results were in line with those of the first outcome examined. Although the strength of the association changed marginally, the results were essentially unchanged (Table 5). However, there were some differences. Indeed, population density and nurses and midwives showed no significant association; however, the literacy rate was moderately negatively associated with vaccination coverage.

**Table 5:** Odds of variables for a percentage of the population vaccinated with at least one dose, both raw (first column) and adjusted (second column).

	**Percentage of the population vaccinated with at least one dose**		**Percentage of the population vaccinated with at least one dose—adjusted**	
	**Odds**	**CI 95%**	**Odds**	**CI 95%**
Median age	1.070	0.995–1.150	1.072	0.996–1.153
Total population	1.132	1.086–1.181	1.118	1.069–1.170
Population density	1.015	0.975–1.056	1.020	0.980–1.062
COVID-19-specific mortality	0.973	0.935–1.012	0.974	0.936–1.014
GDP per capita	1.311	1.168–1.473	1.343	1.192–1.513
Gini Index	1.142	1.101–1.184	1.137	1.095–1.179
Greenberg Index	0.926	0.893–0.960	0.925	0.892–0.958
Literacy rate	0.902	0.843–0.966	0.907	0.847–0.971
Presence of a predominant religion	0.952	0.921–0.985	0.956	0.924–0.990
Density of the road network	0.970	0.902–1.044	0.912	0.820–1.015
Access to electricity	1.433	1.356–1.515	1.427	1.350–1.509
Civil liberties	0.891	0.854–0.929	0.899	0.861–0.939
Political stability and absence of violence/terrorism	1.264	1.196–1.336	1.260	1.192–1.332
Type of health system (public or private)	0.949	0.915–0.984	0.946	0.912–0.981
Health personnel—physicians	1.057	1.009–1.106	1.052	1.004–1.102
Health personnel—nurses and midwives	1.004	0.949–1.064	1.002	0.946–1.061

## 4. Discussion

The aim of this study was to assess possible determinants associated with vaccination coverage for COVID-19. Two outcomes were identified as indicators of vaccination coverage: the percentage of the population vaccinated with at least one dose and the total doses administered per 100 residents.

Analyzes were conducted to test the hypothesis that vaccination coverage could be partly explained by 16 variables in multiple fields—sociodemographic, cultural, infrastructural, economic, political, and health system performance.

Our results showed significant associations between the outcomes and several variables. The determinants were more impactful in determining the doses administered per 100 residents rather than increasing the vaccination coverage rate.

Surprisingly, the distribution of vaccine doses did not influence vaccination coverage at the country level as the raw outcomes were not dissimilar to their adjusted counterparts. This result does not underestimate the need for allocating COVID-19 vaccine doses among countries in an equitable manner and with timely delivery of total COVID-19 vaccine but recommemds that other variables can explain the outcomes.

Consistent with recent literature (45–47) on COVID-19 suggesting that, in general, the wealthier a country is, the higher the vaccination rate is (45, 48), and in line with a World Health Organization analysis reporting that high-income countries had the highest potential of achieving ≥90% national coverage of certain vaccinations (49), our results showed an association between GDP and vaccination coverage. It is realistic to assume that countries with a higher GDP per capita can achieve higher vaccination coverage due to better access to healthcare in developed countries than in developing and resource-restricted settings.

Unexpectedly, the Gini Index was positively associated with outcomes (~15% more likely to increase outcomes): the higher the index and, hence, the inequality in income distribution are, the more the vaccination coverage and doses administered per resident increased. This finding is apparently in contrast with expectations that, if there is strong inequality in the population, large groups of the population are denied the right to healthcare through an unequal access to health services (50–53). Analyzing an epochal and non-ordinary situation such as the COVID-19 pandemic, the availability and gratuitousness of a primary good, such as a vaccine, resulted in the world population having access (54, 55), perhaps for the first time, to preventive services useful to the global public health, even where there was a low GDP per capita and a high Gini Index. It is possible that, in these countries where inequalities were greatest, the free availability of the vaccine promoted population adherence.

It is remarkable that access to electricity was shown to be the determinant with the greatest relative weight; indeed, access to electricity was 62.5% more likely to impact total doses administered than the other variables (43.3% additional likelihood regarding the percentage of population vaccinated). In contrast, road network density did not affect COVID-19 vaccination. These results suggest that the vaccination campaign was more strongly conditioned by the storage of vaccines than by the absence of transportation and geospatial access issues. A previous study revealed that low-income countries sometimes had to refuse vaccine doses due to imminent drug expiration and the impossibility of storing them (56–58), and the WHO explained that minor benefits for vaccine doses distributed to poorer countries can be explained by the limited availability of cold-chain equipment, low warehousing or storage capacities, and a lack of human resources. Due to variations in supply chain readiness, COVAX-eligible countries and territories may not receive their allocation from the COVAX facility until minimum conditions are met (54).

Both political variables were shown to be important determinants of COVID-19 vaccination, albeit in opposite ways. Political stability and the absence of violence/terrorism positively impacted vaccination, consistent with the literature (42, 59, 60). This result might be explained by the fact that the political stability of a country is crucial for regulating the vaccination campaign, communicating the content congruent with government action to citizens, and effectively organizing mass vaccination. Interestingly, civil liberties were one of the determinants of COVID-19 vaccination that had the most negative impact. As identified in a previous study, countries with the greatest protection of civil liberties have lower COVID-19 vaccination coverage (43). A possible explanation is that vaccination, often mandatory, to protect the population's life and health is weighed against individuals' bodily freedom, which includes the right to decline treatment options (61). While people adhering to vaccination laws have accepted the notion of collective welfare, others prioritize individual freedoms over the common good (41). This result is in contrast with a common thought. Indeed, COVID-19 vaccination, individually and especially at the population level, increases people's safe freedom of movement, association, work, and school attendance and contributes to restoring normalcy (62) Choosing vaccination is based partly on the expected gain in freedom (i.e., lessening of limitations) and in unnecessary activities, allowing individuals to travel freely, attend political gatherings, and attend religious services. It is realistic to assume that fears of serious side effects and concerns that the vaccines have not been adequately tested, a lack of trust, social norms, exposure to rumors and myths undermining confidence in vaccines, and failure by some healthcare providers to counter these myths and provide evidence-informed advice play a role in hesitating or refusing to get vaccinated (63). Moreover, it is reasonable to consider the possible influence of the “exceptional” vaccination campaign for COVID-19 on the occurrence of vaccine confidence. The literature has shown a link between vaccine regulation and mistrust among unvaccinated and vaccinated individuals due to the perception of measures to regulate immunity as coercive (64).

Predominantly, public healthcare systems were negatively associated with two outcomes. This result may suggest that public systems perform worse than private systems. Even if several policy efforts must be made to reduce vaccine hesitancy in public healthcare systems (65, 66), an interrelationship among factors may explain this result. Private healthcare systems opted for more profitable treatment avenues, under which hospitals, physicians, and other providers get paid more to treat a disease than prevent it (67). Due to high costs, private systems are inaccessible to most of the population. It is reasonable to assume that, faced with remuneration being much higher for inpatient and outpatient services than for preventive services, a broader segment of the population would be willing to receive an approved vaccine if offered free of charge, considering the vaccine as a means to avoid potential unaffordable expenses due to a possible serious illness requiring hospitalization or outpatient services (68–70). Instead, where health is viewed as a right, and therefore, there is a public health system, people would have more freedom in vaccination choices because they would know that if they became ill, they would be guaranteed treatment, regardless of income and vaccination choice.

Regarding healthcare personnel, these indicators had surprisingly little weight in increasing the likelihood of vaccination, with a clear gap in the favor of physicians, perhaps because there was a greater shortage of physicians than other healthcare personnel worldwide, especially in low- and lower-middle-income countries (71). Thus, owing to the significant weight of these countries, an increase in the proportion of physicians per inhabitant could result in a significant increase in vaccination coverage, albeit not a dramatic one. Consistent with the literature (72–75), this result also supports the hypothesis that physicians, especially primary care physicians, play a critical role in ensuring vaccine acceptance, especially in resource-limited and vaccine-hesitant regions, potentially through counseling and building local community trust and partnerships before vaccines become available.

Surprisingly, and in contrast with the current evidence (46, 76), this study showed the absence of a significant association between COVID-19 mortality and median age. Although it is reasonable to assume that, in countries with higher mortality rates, which are also the countries with higher median age, there is a greater perception of disease severity and age-related risk, and the results were discouraging. Because people's trust in the healthcare system is crucial to obtain compliance with prevention policies, the lack of credibility of healthcare systems is partly responsible for vaccine hesitancy. The phenomenon involves, among other causes, the attribution of negative values (corruption, absence of transparency, and autonomy) to researchers and health authorities, and even governments, because of their relationship with pharmaceutical companies that produce and sell vaccines. Such attribution of negative values undermines the grounds of public trust and diminishes credibility; thus, the evidence provided on vaccine safety is immediately dismissed as tainted (77). Recent health communication studies have shown that the public looks for and receives information about vaccines not from public health officials and practitioners but from friends, celebrities, and social media, and these non-health-professional sources are perceived as credible. Public health officials and practitioners' efforts to correct fake claims about vaccines fail to address the public's main concerns, namely, fears and worries about adverse side effects (78–82).

This phenomenon is a public health issue that implies, potentially, that some COVID-19 deaths could have been avoided by vaccination but instead will likely continue to cause harm to humans beyond economic concerns.

Population density increased only the total doses administered per resident and not the percentage of the population vaccinated. One plausible explanation is that an increase in population density reduces logistical problems and would therefore facilitate access to preventive services for those who adhere to vaccination but did not affect the proportion of the population unwilling to vaccinate. Although the evidence shows that continuing transmission is associated with areas of high population density (83) and fears that are directly related to the physical health of oneself or loved ones are associated with a higher acceptance of a vaccination that promises to reduce the probability of those negative outcomes, this evidence does not seem to predict wider public health compliance (84, 85). It is more difficult to explain the association between the total population and the two outcomes. Further investigations must be conducted to assess whether more pressing vaccination campaigns and policy efforts to improve healthcare access have been adopted in the more populous countries or to evaluate the hypotheses of the literature according to which a role could be played by conformism understood as the tendency of people to adopt to some behaviors, beliefs, or other learned traits conditionally on others having adopted it. Adopting this perspective (86), the increase in population may have led to an increase in the proportion of people adopting the most prevalent population behavior, vaccinating themselves despite a conflict with their own ideas (87).

The cultural indicator findings were peculiar. In contrast to the previous literature concerning routine immunizations (88–92), the literacy rate was not a determinant of vaccination coverage: some studies assessed the positive association between health literacy (93) and vaccination coverage; however, the literature concerning literacy was scarce. Given this major discrepancy, a subtle difference that could effectively explain the result is worth reporting: literacy differs substantially from health literacy as the ability to read and the ability to correctly understand health information are two very different matters. Therefore, introducing a programme to increase basic scientific knowledge (e.g., regarding vaccines) into the primary school curriculum would be appropriate. Nevertheless, an increase in the literacy rate is likely to promote vaccinations in less developed countries as the population is less literate in these countries; hence, these individuals have fewer tools to interpret health information correctly. Studies have shown that religion influences decision-making regarding preventive behaviors (94) and significantly influences people's vaccination decisions (95, 96). Similar to previous studies (97, 98), this study shows that the presence and growing prevalence of a predominant religion relates to an increased likelihood of reduced vaccination coverage (97, 98), suggesting that religions may lead to mistrustful or anti-scientific stances toward COVID-19 vaccination and that religiosity may influence vaccine-related attitudes and decisions. Because the role of religious groups and faith leaders has been identified as critical in evaluating vaccination attitudes (99), dialogue with these religions should be considered (100).

Finally, as expected and consistent with past recent literature (101), the presence of linguistic and thus the ethnocultural fragmentation of a country lead to an increased likelihood of reduced vaccination coverage, negatively affecting both outcomes, not markedly but still significantly. Such a finding may have different explanations: on the one hand, ethnic and cultural differences often reflect religious differences, as stated previously. On the other hand, linguistic and ethnic fragmentation may have been an obstacle to central government action or at least an important barrier to communicating and conveying information and reasons related to the COVID-19 vaccination campaign.

Moreover, it is important to mention the possible influence of recent great milestones achieved in low and lower-middle-income countries, such as the decline in measles mortality rates or the certification of the WHO African Region as free of wild poliovirus, on increasing vaccine hesitancy, reducing vaccination coverage in general and thus COVID-19 vaccine intake. Nevertheless, previous literature has identified misinformation in mainstream media and social networks and a lack of an effective communication policy by scientific and political authorities—closely linked to a country's political stability and civil liberties—regarding COVID-19 vaccines, which could create distrust within communities and compromise vaccination programmes (102).

The current study has several limitations, mainly related to the database used, and are common to large database studies. The incompleteness of the data limits the use of such databases. Consequently, it has not been possible to consider all the countries of the world, only those for which data related to the analyzed variables were available. Similarly, the set of variables used did not encompass all variables that could affect vaccination coverage, such as the density of railways, trust in governments or containment measures in response to the COVID-19 outbreak, and the stringency index, due to the lack of information on most countries. In addition, data for each variable are gathered for the most recent year available. Moreover, the database provides data at the country level and inevitably conceals the effects of large variations in terms of cultural and sociodemographic variables and other factors, such as how vaccine doses were allocated and distributed within countries after delivery. This study is a pioneer in the search for the identification of determinants of vaccination coverage in different countries using a variety of status-related variables in multiple fields. This approach is supported by specialized literature (103, 104) and overcomes the limitations of many studies focusing on a few indicators or only sociodemographic indicators (41, 47, 48). Finally, the study's results must be considered with caution due to potential differences in data collection methods across countries and the potential underreporting of certain variables in some settings.

## 5. Conclusion

This study is the first to analyze different categories of indicators that could influence COVID-19 vaccination to comprehend a very complex reality, identifying some as determinants. The multivariate analysis made it possible to reliably establish the degree of association of each variable with the outcomes, thus obtaining results as error-free as possible, although not always easy to interpret.

The study covered 205 countries and assessed 16 explanatory variables, and the results should be contextualized in the different states according to their peculiarities. From a global perspective, improving economic conditions and ensuring access to electricity for all peoples, without borders, would contribute substantially to ensuring free access to vaccines.

The results offer policymakers, governments, and supranational authorities a range of determinants, focusing on which, through prioritization strategies, intervention for a mass vaccination campaign in a pandemic context would yield the greatest returns. Clearly, the determinants can weigh differently in different countries, and policymakers should measure their interventions in compliance with the most impactful determinants of vaccination coverage in each specific country.

Obviously, the imperative would be to implement changes now to improve the ongoing COVID-19 vaccination campaign instead of waiting for the next pandemic. Perhaps aiming for less inequality between nations is an ideal approach for pursuing a common good in terms of public health.

## Data availability statement

The data analyzed in this study is subject to the following licenses/restrictions. Data are available upon reasonable request addressed to corresponding author. Requests to access these datasets should be directed at: alberto.peano@unito.it.

## Author contributions

AP contributed to the conceptualization, investigation, methodology, resources, visualization, and writing (original draft, review, and editing) of this manuscript. GP contributed to the formal analysis and writing of the original draft of this article. MG contributed to the conceptualization, methodology, project administration, supervision, and writing (original draft, review, and editing) of this manuscript. All authors contributed to the article and approved the submitted version.
