# Supplementary material for: Determinants of COVID-19 vaccination worldwide: WORLDCOV, a retrospective observational study
Source: Front Public Health. 2023 Aug 31;11:1128612. doi: 10.3389/fpubh.2023.1128612 (PMC10501313; doi:10.3389/fpubh.2023.1128612)
Supplement: Supplementary file 1 [file Table_1.docx]

**Supplementary file 1.** Countries and territories included in the study sorted alphabetically according to ISO 3166 code.

Aruba

Afghanistan

Angola

Albania

Andorra

United Arab Emirates

Argentina

Armenia

Antigua and Barbuda

Australia

Austria

Azerbaijan

Burundi

Belgium

Benin

Burkina Faso

Bangladesh

Bulgaria

Bahrain

Bahamas, The

Bosnia and Herzegovina

Belarus

Belize

Bermuda

Bolivia (Plurinational State of)

Brazil

Barbados

Brunei Darussalam

Bhutan

Botswana

Central African Republic

Canada

Switzerland

Chile

China

Côte d'Ivoire

Cameroon

Democratic Republic of Congo

Congo

Colombia

Comoros

Cabo Verde

Costa Rica

Cuba

Curacao

Cayman Islands

Cyprus

Czech Republic

Germany

Djibouti

Dominica

Denmark

Dominican Republic

Algeria

Ecuador

Egypt

Spain

Estonia

Ethiopia

Finland

Fiji

France

Faeroe Islands

Micronesia (Federated States of)

Gabon

United Kingdom of Great Britain and Northern Ireland

Georgia

Ghana

Gibraltar

Guinea

Gambia, The

Guinea-Bissau

Equatorial Guinea

Greece

Grenada

Greenland

Guatemala

Guyana

Hong Kong SAR, China

Honduras

Croatia

Haiti

Hungary

Indonesia

Isle of Man

India

Ireland

Iran (Islamic Republic of)

Iraq

Iceland

Israel

Italy

Jamaica

Jordan

Japan

Kazakhstan

Kenya

Kyrgyzstan

Cambodia

Kiribati

Saint Kitts and Nevis

Republic of Korea

Kuwait

Lao People's Democratic Republic

Lebanon

Liberia

Libya

Saint Lucia

Liechtenstein

Sri Lanka

Lesotho

Lithuania

Luxembourg

Latvia

Macao SAR, China

Morocco

Monaco

Republic of Moldova

Madagascar

Maldives

Mexico

Marshall Islands

North Macedonia

Mali

Malta

Myanmar

Montenegro

Mongolia

Mozambique

Mauritania

Mauritius

Malawi

Malaysia

Namibia

New Caledonia

Niger

Nigeria

Nicaragua

Netherlands

Norway

Nepal

New Zealand

Oman

Pakistan

Panama

Peru

Philippines

Palau

Papua New Guinea

Poland

Portugal

Paraguay

West Bank and Gaza

French Polynesia

Qatar

Romania

Russian Federation

Rwanda

Saudi Arabia

Sudan

Senegal

Singapore

Solomon Islands

Sierra Leone

El Salvador

San Marino

Somalia

Serbia

South Sudan

Sao Tome and Principe

Suriname

Slovak Republic

Slovenia

Sweden

Eswatini

Seychelles

Syrian Arab Republic

Turks and Caicos Islands

Chad

Togo

Thailand

Tajikistan

Turkmenistan

Timor-Leste

Tonga

Trinidad and Tobago

Tunisia

Turkey

Taiwan

United Republic of Tanzania

Uganda

Ukraine

Uruguay

United States of America

Uzbekistan

Saint Vincent and the Grenadines

Venezuela (Bolivarian Republic of)

Viet Nam

Vanuatu

Samoa

Kosovo

Yemen

South Africa

Zambia

Zimbabwe
